# Glyphosate and AMPA Induce Alterations in Expression of Genes Involved in Chromatin Architecture in Human Peripheral Blood Mononuclear Cells (In Vitro)

**DOI:** 10.3390/ijms22062966

**Published:** 2021-03-15

**Authors:** Ewelina Woźniak, Edyta Reszka, Ewa Jabłońska, Jaromir Michałowicz, Bogumiła Huras, Bożena Bukowska

**Affiliations:** 1 Department of Biophysics of Environmental Pollution, Faculty of Biology and Environmental Protection, University of Lodz, Pomorska 141/143, 90-236 Lodz, Poland; ewelina.wozniak@umed.lodz.pl (E.W.); jaromir.michalowicz@biol.uni.lodz.pl (J.M.); 2 Laboratory of Tissue Immunopharmacology, Department of Internal Diseases and Clinical Pharmacology, Medical University of Lodz, Kniaziewicza 1/5, 91-347 Lodz, Poland; 3 Department of Molecular Genetics and Epigenetics, Nofer Institute of Occupational Medicine, Teresy 8, 91-348 Lodz, Poland; Edyta.Reszka@imp.lodz.pl (E.R.); Ewa.Jablonska@imp.lodz.pl (E.J.); 4 Łukasiewicz Research Network, Institute of Industrial Organic Chemistry, Annopol 6 Str, 03-236 Warsaw, Poland; bogumila.huras@ipo.lukasiewicz.gov.pl

**Keywords:** glyphosate, AMPA, chromatin structure, epigenetics, human peripheral blood mononuclear cells

## Abstract

We have determined the effect of glyphosate and aminomethylphosphonic acid (AMPA) on expression of genes involved in chromatin architecture in human peripheral blood mononuclear cells (PBMCs). The cells were incubated with glyphosate and AMPA in the concentrations ranging from 0.5 to 100 μM and from 0.5, to 250 μM, respectively. The expression profile of the following genes by quantitative Real-Time PCR was evaluated: Genes involved in the DNA methylation (*DNMT1*, *DNMT3A*) and DNA demethylation process (*TET3*) and those involved in chromatin remodeling: genes involved in the modification of histone methylation (*EHMT1*, *EHMT2*) and genes involved in the modification of histone deacetylation (*HDAC3*, *HDAC5*). Gene profiling showed that glyphosate changed the expression of *DNMT1*, *DMNT3A*, and *HDAC3*, while AMPA changed the expression of *DNMT1* and *HDAC3*. The results also revealed that glyphosate at lower concentrations than AMPA upregulated the expression of the tested genes. Both compounds studied altered expression of genes, which are characteristic for the regulation of transcriptionally inactive chromatin. However, the unknown activity of many other proteins involved in chromatin structure regulation prevents to carry out an unambiguous evaluation of the effect of tested xenobiotics on the studied process. Undoubtedly, we have observed that glyphosate and AMPA affect epigenetic processes that regulate chromatin architecture.

## 1. Introduction

Glyphosate was discovered to possess herbicidal properties in 1970. Since that time, the use of glyphosate-based herbicides (GBHs) has increased 100-fold [1] with annual application globally in the range of 0.6–1.2 million tons, making GBHs the most widely used herbicides [2,3]. An increasing use of GBHs is mostly attributed to the development of genetically modified crops (GMOs) along with glyphosate’s action as a broad-spectrum, nonselective herbicide [4,5]. As glyphosate is extensively used in agricultural food production, the human population is exposed both occupationally and environmentally (mainly through the consumption of its residues in food) to its actions.

Aminomethylphosphonic acid (AMPA) is the major product of environmental transformation of glyphosate [6]. Glyphosate and AMPA were selected as priority substances in the Human Biomonitoring for Europe initiative [7]. There is insufficient evidence to indicate whether glyphosate is metabolized to AMPA in humans, or whether AMPA present in human samples mainly derives from AMPA residues in cultivated food products [8,9].

Many scientific studies have indicated the presence of glyphosate in the human body. For instance, glyphosate was determined in the blood of people who were not directly exposed to this herbicide (0.435 ± 0.167 μM) [10] and humans occupationally exposed (0.05–0.5 mM) [11], as well as in human urine of the Swedish population of young adults, where the median and maximum concentrations of this herbicide were calculated to be 0.03, and 3.39 μg/L, respectively [12].

Soukup et al. [9] observed (a study conducted in Germany with 301 participants) significant, positive associations between urinary glyphosate excretion and consumption of pulses, or urinary AMPA excretion and mushroom intake. They suggested that despite the widespread use of glyphosate, the exposure of the German population to glyphosate and AMPA was very low. Based on the current risk assessment of glyphosate by European Food Safety Authority (EFSA), such exposure levels are not expected to pose any risk to human health.

Exposure to environmental toxicants can lead to heritable or reversible modifications that can affect gene expression without changing DNA structure, otherwise known as epigenetic changes [13]. The epigenetic processes, include DNA methylation, histone modifications, noncoding RNA regulation, chromatin structure remodeling, and RNA methylation. The knowledge about the ability of glyphosate and other environmental xenobiotics to epigenetic modifications in humans is of great importance.

Recently, Kubsad et al. [14] observed that glyphosate promoted the epigenetic transgenerational inheritance of disease and pathology through germline (i.e., sperm) epimutation in female rats. Negligible pathology was noticed in the F0 and F1 generations, while a significant increase in pathology and disease was noted in the F2 generation grand-offspring and F3 generation great-grand-offspring. Moreover Ben Maamar et al. [15] showed that glyphosate caused the epigenetic transgenerational inheritance of pathology and disease in subsequent great grand offspring (F3 generation) of rats. Those studies have shown that the differential histone retention in sperm appears to have a role in epigenetic transgenerational inheritance.

Recent findings have suggested that glyphosate and AMPA affect changes in the human epigenome, associated with alterations in global methylation [16,17,18,19]. Furthermore our previous studies [20,21] had shown that glyphosate and AMPA significantly altered global DNA methylation in peripheral blood mononuclear cells (PBMCs), methylation in the promoter regions of tumor suppressor genes as well as expression of major cell cycle drivers and apoptosis. However, it should be emphasized here that in vitro studies on the effect of glyphosate on PBMCs apoptosis [22] showed that this herbicide only at a very high concentration of 0.5 mM caused apoptotic alterations in the tested cells. Therefore, the above-mentioned result suggests that alterations of DNA methylation patterns do not have to influence specific metabolic effects until exposure to high doses or cumulative long-term exposure of humans to glyphosate.

Chromatin architecture may be affected by various exposures that can directly contact DNA or transcription factors and various binding elements to cause epigenetic changes [23]. The chromatin condensation level can significantly influence the expression of genes and the sensitivity of the DNA to injury. Heterochromatin is resistant to DNA-damaging agents, while euchromatin has increased sensitivity to damage [24,25]. In our previous study [26], glyphosate and AMPA had been shown to induce DNA single strand-breaks (double strand breaks—only glyphosate) and cause purines and pyrimidines oxidation in PBMCs.

Post-translational modifications of both histones within the chromatin and the DNA itself are diverse ways in which the chromatin structure can be remodeled. These modifications are controlled and correspond to various factors, for example, cell cycle. We [20,21] have shown that glyphosate and AMPA changed the methylation pattern of the *P16* and *TP53* suppressor gene promoters, while glyphosate additionally affected the methylation of the *P21* protooncogene promoter. Gene profiling showed that glyphosate altered the expression of genes involved in cell cycle regulation: *CCND1*, *P16,* and *TP53*, while AMPA affected the expression only of *CCND1*.

Because glyphosate and AMPA influenced the above-mentioned factors determining the chromatin state, we decided to determine expression of genes involved in the DNA methylation (*DNMT1*, *DNMT3A*) and DNA demethylation process (*TET3*), as well as chromatin remodeling: analysis of expression of genes involved in the modification of histone methylation (*EHMT1*, *EHMT2*) and expression of genes involved in the modification of histone deacetylation (*HDAC3*, *HDAC5*). 

Specified genes were selected because preliminary reports have shown changes in the expression of these genes in PBMCs and their key role in the establishment of chromatin architecture.

The human cells were incubated with glyphosate in concentrations ranging from 0.5 to 100 μM and with AMPA in concentrations ranging from 0.5 to 250 μM, which did not induce cytotoxic effects on PBMCs viability.

## 2. Results

### 2.1. Analysis of Expression of Genes Involved in the DNA Methylation (DNMT1, DNMT3A) and DNA Demethylation Process (TET3)

**Figure 1:**
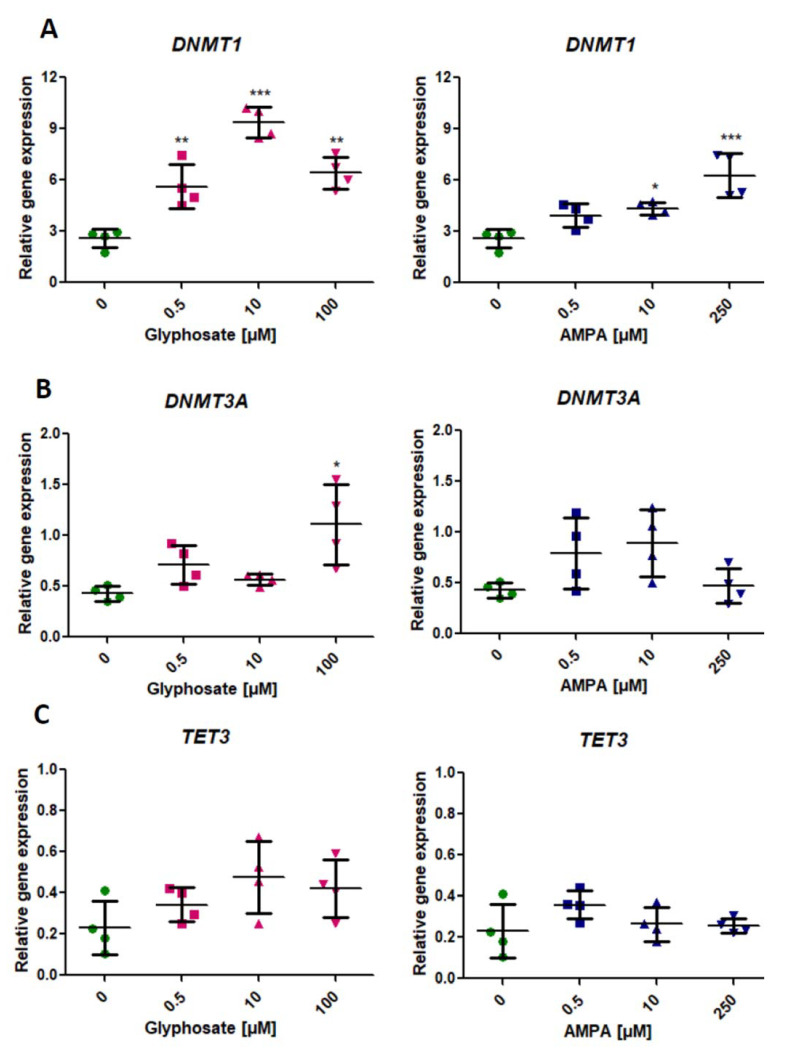
Expression of genes involved in DNA methylation—*DNMT1* (**A**), *DNMT3A* (**B**), and DNA demethylation—*TET3* (**C**) process in human PBMCs incubated with glyphosate (0.5–100 µM) and AMPA (0.5–250 µM) for 24 h. Mean ± SD was calculated for four individual experiments (four blood donors). Statistically different from control at * *p* < 0.05; ** *p* < 0.01 and *** *p* < 0.001. Statistical analysis was conducted using one-way ANOVA and a posteriori Tukey test.

We found a statistically significant (*p* < 0.05, one-way ANOVA) increase of *DNMT1* expression in PBMCs treated with all concentrations of glyphosate and two concentrations of AMPA (10 µM and 250 µM) for 24 h (Figure 1A). 

Glyphosate induced a significant increase in *DNMT3A* expression only at its highest concentration of 100 µM (*p* < 0.05, one-way ANOVA). We did not observe statistically significant changes in *DNMT3A* expression in PMBCs treated with AMPA (Figure 1B). 

Treatment of PBMCs with glyphosate and AMPA did not affect the expression of *TET3* (Figure 1C).

### 2.2. Analysis of the Expression of Genes Involved in the Modification of Histone Methylation (EHMT1, EHMT)

**Figure 2:**
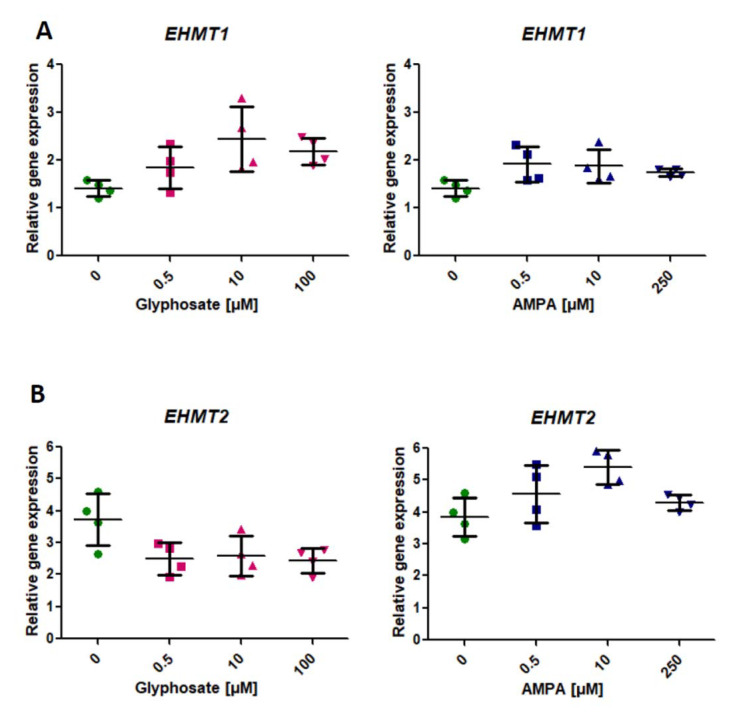
Expression of genes involved in histone methylation—EHMT1 (**A**), EHMT2 (**B**) in human PBMCs incubated with glyphosate (0.5–100 µM) and AMPA (0.5–250 µM) for 24 h. Mean ± SD was calculated for four individual experiments (four blood donors). Statistical analysis was conducted using one-way ANOVA.

We did not observe any statistically significant changes in *EHMT1* and *EHMT2* expression in PBMCs treated for 24 h with glyphosate and AMPA (*p* > 0.05, one-way ANOVA) (Figure 2A,B). However, after 24-h treatment of PBMCs with glyphosate, an upward trend in the expression of EHMT1 was noticed (Figure 2A). Similarly, incubation of the cells with AMPA showed an upward trend in the expression of *EHMT2* (Figure 2B).

### 2.3. Analysis of Expression of Genes Involved in the Modification of Histone Deacetylation (HDAC3, HDAC5) (HDAC3, HDAC5)

**Figure 3:**
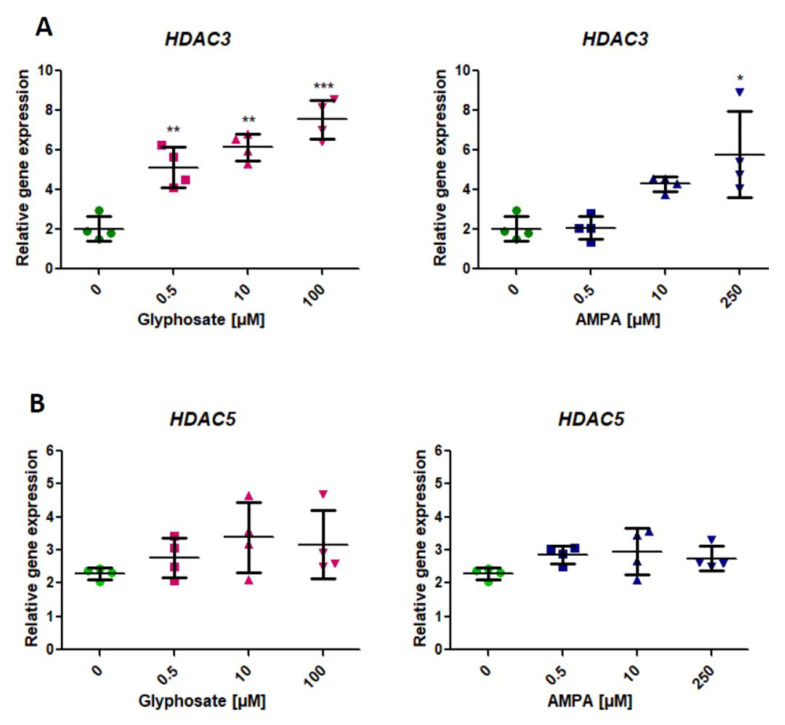
Expression of genes involved in the histone deacetylation—*HDAC3* (**A**) and *HDAC5* (**B**) in human PBMCs incubated with glyphosate (0.5–100 µM) and AMPA (0.5–250 µM) for 24 h. Mean ± SD was calculated for four individual experiments (four blood donors). Statistically different from control at * *p* < 0.05; ** *p* < 0.01 and *** *p* < 0.001. Statistical analysis was conducted using one-way ANOVA and a posteriori Tukey test.

We found a statistically significant (*p* < 0.05, one-way ANOVA) increase of *HDAC3* expression in PBMCs treated with glyphosate from its lowest concentration of 0.5 μM for 24 h (Figure 3A). AMPA induced a significant increase in *HDAC3* expression only at its highest concentration of 250 µM (*p* < 0.05, one-way ANOVA) (Figure 3A).

We did not observe any statistically significant changes in *HDAC5* expression in PMBCs treated with the tested xenobiotics (Figure 3B).

## 3. Discussion

In this study, for the first time, we investigated the indirect effect of glyphosate and its metabolite—aminomethylphosphonic acid (AMPA) on epigenetic modifications associated with the regulation of chromatin structure, through the analysis of the expression patterns of genes involved in DNA and histone regulation in PBMCs in vitro model.

Chromatin condensation and decondensation is a complex process that depends on many factors, including various protein complexes, histone variants, and biochemical modifications. DNA methylation is regulated by a number of enzymes, including DNA methyltransferase (DNMT) and histone deacetylase (HDAC). DNMT1 works as a maintenance methyltransferase, preferably methylating the fifth position of the cytosine residues), ensuring the same methylation pattern during DNA replication and cell division, while DNMT3A enzymes, known as de novo methyltransferases, work to establish new methylation patterns of hypomethylated DNA necessary for tissue-specific differentiation during development [27,28,29]. In silico evaluation of pesticide action as potential modulators of human DNA methyltransferases, showed that rodenticides (flocoumafen, brodifacoum, difenacoum) are potential DNMT (for both DNMT1 and DNMT3A) ligands, and therefore, may modulate DNA methylations [30]. It must, however, be kept in mind that rodenticides have a different mechanism of action than glyphosate and AMPA.

We have found an increased *DNMT1* expression in response to treatment of PBMCs with glyphosate at all its concentrations and AMPA at 10 µM and 250 µM. Further analysis of enzymes, known as de novo methyltransferases revealed an increased expression of the *DNMT3A* gene in glyphosate-treated tested cells (100 μM). Our analyses have not shown any changes in *DNMT3A* expression in PBMCs incubated with AMPA. Just recently, changes in the DNA methylation machinery due to glyphosate exposure were identified in the fish model of Japanese medaka (*Oryzias latipes*), where upregulation of *Dnmt1*, *Dnmt3a* gene expression was shown [31]. In addition other pesticides (with different mechanism of action than glyphosate) extensively used around the world, revealing similar effects. For instance, treatment of the human breast cancer cell line, MCF-7 for 12 h and 24 h with endosulfan at 10 μM led to significant upregulation of both *DNMT1* and de novo *DNMT3A* and *DNMT3B*. Moreover, the total intracellular histone deacetylase (HDAC) activity was found to be significantly increased, which was correlated with the upregulation of class I HDACs (HDAC 1 and HDAC3), while no significant alteration in the other HDAC classes was observed in MCF-7 cells [32]. 

Our analysis of the expression of histone deacetylases revealed an increased expression of *HDAC3* in PBMCs exposed to glyphosate at its all tested concentrations (0.5–100 μM) and AMPA at its highest concentration of 250 μM. However, no statistically significant changes in the expression level of *HDAC5* have been noted.

Chromatin plays an essential role in the activation and inhibition of gene transcription by regulating the availability of transcription factor (TF) binding. Increased *DNMT1* and *HDAC3* activity contributes to the condensation of chromatin, preventing TF attachment, resulting in inhibition of transcription. It has been documented that such a phenomenon accompanies the process of neoplastic transformation and relates primarily to silencing suppressor gene expression [33,34,35,36,37,38]. 

**Figure 4:**
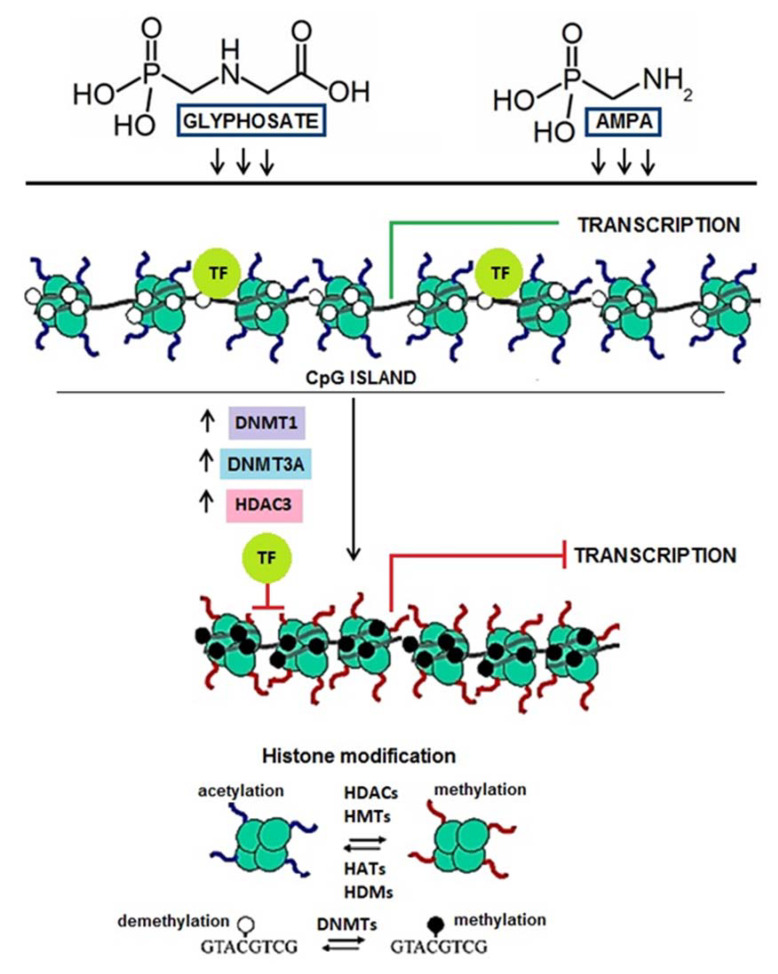
The potential impact of glyphosate and AMPA on chromatin structure. Chromatin plays a crucial role in gene activation and inhibition by regulating the accessibility of transcription factor (TG) binding. Methyl groups are coupled to cytosines by the family of DNA methyltransferases (DNMTs), where DNMT1 is the main maintenance enzyme and the DNMT3A branch of the family is mostly responsible for de novo methylation.

We have observed that glyphosate and AMPA increased expression of *DNMT1* and *HDAC3* genes. Consequently, it can lead to high activity of DNMT1 and HDAC3 and finally to chromatin condensation, which prevents TFs binding and inhibition of transcription (Figure 4). 

Ten-eleven translocations (TET) are other enzymes active in DNA methylation, which oxidize 5-methylcytosine (5-mC) to 5-hydroxymethylcytosine (5-hmC), leading to DNA demethylation by removing 5-mC from the genome [39,40,41]. Duforestel et al. [17] used nonneoplastic MCF10A cells in a repeated glyphosate exposure pattern over 21 days. They observed that glyphosate triggered a significant increase in the activity of ten-eleven translocation (TET3) enzyme and could affect breast cells to tumorigenesis via epigenetic reprogramming, which occurred through TET3-mediated global and local DNA hypomethylation. In the aforementioned studies on the fish model of Japanese medaka (*Oryzias latipes*), glyphosate also caused upregulation of *Tet1* and *Tet3* gene expression [31]. Similar results were obtained in the rodent model. A two-year study on rats given 0.1 ppb of Roundup (50 ng/L of glyphosate equivalent) via drinking water (daily intake of 4 ng/kg bw/day of glyphosate) was conducted and showed an increased expression of *Dnmt3a* and *Tet3* gene [42] Nevertheless, our analysis has not revealed any changes in the expression level of *TET3* gene in human PBMCs incubated with glyphosate or AMPA.

To date, there is no information regarding the effect of glyphosate on histone modification in human cells. The only previously mentioned study by Mesnage et al. [42] showed the effect of glyphosate on the genes responsible for regulating the structure of chromatin (*Men1*, *Setdb1*, *Suv420h2*, *Dot1l*, *Ehmt1*, *Ehmt2*, *Nsd1*). However, we have not observed any statistically significant changes in *EHMT1* and *EHMT2* gene expression, in human PBMCs incubated with tested xenobiotics.

The main limitation of our work is the lack of measurement of histone acetylation levels upon glyphosate/AMPA treatment. The results would be significantly strengthened if the biological effect of the increase of HDAC3 in PBMCs on histone acetylation could be assessed. Additionally, the information about the potential influence of glyphosate on the activity of the enzymes (rather than their expression) would be important. These studies would show a measurable, potential biological effect of changes in the expression of the studied genes.

## 4. Materials and Methods

### 4.1. Chemicals

*N*-(phosphonomethyl)glycine (glyphosate) (purity 95%), fetal bovine serum (FBS), penicillin/streptomycin, TRIzolTM and primers were bought from Sigma-Aldrich®, (Darmstadt, Germany). Aminomethylphosphonic acid (AMPA) (purity 98%) were synthetized by the Institute of Industrial Organic Chemistry, Łukasiewicz Research Network, Warsaw, Poland. 

RPMI 1640 medium with L-glutamine and lymphocyte separation medium (LSM) (1.077 g/cm^3^) were purchased from Cytogen (Greven, Germany). Transcriptor First Strand cDNA Synthesis Kit and FastStart Essential DNA Green Master were purchased from Roche (Basel, Switzerland). TRIzolTM Reagent was bought from Thermo Fischer Scientific, Waltham, MA, USA. Other chemicals were from Carl Roth (Karlsruhe, Germany) and POCh (Gliwice, Poland) and were of analytical grade.

### 4.2. Cells Isolation

PBMCs were isolated from a leucocyte-buffy coats obtained from blood purchased in Blood Bank in Lodz, Poland. Blood was obtained from four healthy volunteers (aged 18–55), who showed no signs of infection symptoms at the time the blood samples were collected. The investigation was approved by the Bioethics Committee of the University of Lodz No. 1/KBBN-UŁ/II/2017. 

Cell isolation was conducted according to the procedure described by Woźniak et al. [26]. The final PBMCs density used in the experiments (after addition of glyphosate) was 1x10^6^ cells/mL.

### 4.3. Cells Treatment

Glyphosate and AMPA were dissolved in PBS, pH 7.4. The cells were incubated with glyphosate at final concentrations of 0.5; 10; 100 μM and with AMPA at finally concentrations of 0.5; 10; 250 μM for 24 h in four independent experiments on blood samples collected from four donors. PBS (in which xenobiotics were dissolved) was added to the control samples. Cells suspension from individual donor (396 µL) was incubated with 4 µl of PBS (control sample), 4 µL of glyphosate or 4 µL of AMPA (blood samples from four donors were used for all control or glyphosate/AMPA treatments).

The concentrations of glyphosate and AMPA used in this study corresponded to their levels determined in humans environmentally exposed. No statistically significant change in cell viability were observed as a result of PBMCs treatment with glyphosate or AMPA at selected (above mentioned) concentrations as previously presented by Woźniak et al. [26]. Obligatory, in each series of probes, the viability of the tested cells was determined by Trypan Blue dye exclusion test.

During incubation, the cells were resuspended in RPMI supplemented with 10% FBS and penicillin/streptomycin solution (50 U/mL) at 37 °C and 5% CO_2_. After incubation, the cells were centrifuged, the tested compounds were discarded, and the cells were resuspended in RPMI medium.

### 4.4. Gene Expression

RNA was extracted with TRIzolTM. RNA samples with a 260/280 nm ratio in the range of 1.8–2.0 were used for further analysis. The isolated RNA was reversely transcribed (Transcriptor First Strand cDNA Synthesis Kit, Roche), and cDNA was quantified by real-time PCR (FastStart SYBR Green Master, Roche, Basel, Switzerland). 

*GAPDH*, *RPL13*, *RPLP0* were used as reference genes, verified for PMBCs. Primers listed in Table 1 were designed by means of Primer-BLAST NCBI—NIH website: https://www.ncbi.nlm.nih.gov/tools/primer-blast/ (accessed on 1 February 2020). The 2^−ΔCt^ method was used to calculate the expression levels of the studied genes and expressed as relative copy number values [43].

### 4.5. Statistical Analysis

Statistical analysis was performed with STATISTICA 13.1 data analysis software (2000 Stat-Soft, Inc., Tulsa, OK, USA). We checked a normality of relative expression distribution using Shapiro-Wilk test as well as the homogeneity of variance by Brown-Fisher test. Statistical analysis was conducted using one-way analysis of variance (ANOVA) followed by Tukey’s post hoc multiple comparison procedure. The difference was considered significant for *p* < 0.05. The individual analysis was performed on blood from four donors, while a single experiment conducted on blood from one donor was repeated twice or thrice depending on the method used.

## 5. Conclusions

Summing up, in this study, gene profiling have shown that glyphosate upregulated the expression of genes involved in the DNA methylation process, i.e., *DNMT1* and *DMNT3A* as well as *HDAC3* genes involved in the deacetylation of histone proteins, while AMPA altered the expression of *DNMT1* and *HDAC3*. It must be noted that glyphosate at lower concentrations than AMPA caused changes in the expression of these genes.

Our results have revealed a potential change in gene expression in human PBMCs exposed to glyphosate and AMPA, but the observed alterations do not prejudge the final regulation of chromatin structure, which is depended on many other factors. More studies are required, including those based on long-term exposure models, to provide deeper insights into the epigenetic effect of glyphosate and its further implications at the cellular level.

## Figures and Tables

**Table 1:** The primers’ sequences used in the Real-Time PCR.

Gene	Primer	Nucleotide Sequence 5′-3′	PCR Product [bp]
*DNMT1*	forward reverse	GTGGAAGCCGGCAAAGC TCCCACTCGAGCCTTCCATA	125
*DNMT3A*	forward reverse	AAGGAGGAGCGCCAAGAG ATCACCGCAGGGTCCTTT	113
*EHMT1*	forward reverse	CCTCGACTCGGAAAAACCCAA AGAGCGCTTATTCTGGTGCT	148
*EHMT2*	forward reverse	CATCGATCGCAACATCACCC GAGCAATCGCCCATCCTTGT	121
*HDAC3*	forward reverse	TGACTCTCTGGGCTGTGATCG CATATTCAACGCATTCCCCATGC	74
*HDAC5*	forward reverse	CTTAGCAAGTGCGAGCGGAT TGTCTAGCTTCTGCCGGTTG	121
*TET3*	forward reverse	CACTAGCTGTACCAACCGCC CAGCCTTTATTTCCACCTCCTTGA	104
**House keeping genes**			
*GAPDH*	forward reverse	AGCCACATCGCTCAGACAC GCCCAATACGACCAAATCC	66
*RPL0*	forward reverse	TCTACAACCCTGAAGTGCTTGAT CAATCTGCAGACAGACACTGG	96
*RPL13*	forward reverse	CAAGCGGATGAACACCAAC TGTGGGGCAGCATACCTC	95

## Data Availability

Not applicable.

## Abbreviations

AMPA	Aminomethylphosphonic acid
*DNMT1*	DNA methyltransferase
*DNMT3A*	DNA methyltransferase 3 alpha
ECHA	European Chemicals Agency
EFSA	European Food Safety Authority
*EHMT1*	Euchromatic histone lysine methyltransferase 1
*EHMT2*	Euchromatic histone lysine methyltransferase 2
GMOs	Genetically modified crops
GBHs	Glyphosate-based herbicides (GBHs)
HBM4EU	Human Biomonitoring for Europe initiative
*HDAC3*	Histone deacetylase 3
*HDAC5*	Histone deacetylase 5
IARC	International Agency for Research on Cancer
PMBCs	Human peripheral blood mononuclear cells
TET1	Tet methylcytosine dioxygenase 1
*TET3*	Tet methylcytosine dioxygenase 3
TF	Transcription factor binding
US EPA	United State Environmental Protection Agency
WHO	World Human Organization
